# Rigid ureteroscopic lithotripsy with a pressure-controlling ureteral access sheath for complex steinstrasse

**DOI:** 10.1186/s12894-024-01501-z

**Published:** 2024-05-28

**Authors:** Zhong Yuming, Yao Lei, Zhai Qiliang, Huang Xin, Kuang Jin, Leming Song, Deng Xiaolin

**Affiliations:** Department of Urology, Ganzhou People’s Hospital, 17 Hongqi Avenue, Ganzhou, 341000 Jiangxi China

**Keywords:** Ureteroscopy, Cavity pressure, Ureteral access sheath, Steinstrasse

## Abstract

**Objective:**

To evaluate the safety and efficacy of rigid ureteroscopic lithotripsy with a pressure-controlling ureteral access sheath (PC-UAS) for complex steinstrasse.

**Methods:**

Thirty-one consecutive patients (male: 18; female: 13) with steinstrasse were enrolled, six of whom had concurrent kidney stones. The mean cumulative stone size was 2.7 ± 1.3 cm. The patients were treated with rigid ureteroscopic lithotripsy using a PC-UAS. The cavity pressure parameters were set as follows: control value at -15 mmHg to -2 mmHg, warning value at 20 mmHg, and limit value at 30 mmHg. The infusion flow rate was set at 150–200 ml/min. A holmium laser (550 μm) was used to powderize the stone at 2.0–2.5 J/pulse with a frequency of 20–30 pulses/s. Analyses included cavity pressure, operative time, stone-free rates, and complications.

**Results:**

Among the 31 patients, 29 were successfully treated with PC-UAS, with nine requiring adjunctive flexible ureteroscopy for stone migration to the kidney. Two procedures were converted to percutaneous nephrolithotomies due to failure of sheath placement. The cavity pressure of all 29 patients was well-maintained below 20 mmHg, with clear vision. The mean operative time was 48.2 ± 17.7 min. No complications, such as ureteral perforation, mucosal avulsion, or hemorrhage, occurred. Two cases of Clavien-Dindo grade I complications occurred. No major complications (Clavien-Dindo grade II–V) occurred. The mean postoperative hospitalization time was 1.7 days. The stone-free rates 1 day and 1 month after surgery were 93.1% and 96.6%, respectively. One patient with residual stones underwent extracorporeal shockwaves.

**Conclusions:**

Rigid ureteroscopic lithotripsy with PC-UAS can effectively control the cavity pressure, shorten the operation time, and improve the efficiency of broken stones, thus reducing the complication rate.

## Introduction

Urolithiasis is a disease with high morbidity and recurrence rates, with an increasing annual incidence [1]. With the development of flexible ureteroscopes and holmium lasers, an increasing number of doctors are attempting to use flexible ureteroscopy to treat kidney stones > 2 cm; however, large amounts of crushed stone often need to be slowly excreted after surgery, which also leads to steinstrasse formation. Surgical management of steinstrasses has always been difficult, especially for complex steinstrasses [2]. Percutaneous nephrolithotomy (PCNL), rigid ureteroscopy, and flexible ureteroscopy have been used; however, these are associated with certain risks: PCNL is associated with a high risk of trauma and bleeding; the repeated entry and exit of a rigid ureteroscope into the ureter can easily cause ureteral injuries; and traditional flexible ureteroscopy has low efficiency in stone removal, and has a high risk of complications such as infection and ureteral stenosis [3]. Therefore, researchers have reported the use of a vacuum-assisted ureteral access sheath (UAS) to improve the safety and efficiency of complex steinstrasses [4]. However, cavity pressure monitoring and control are still not possible and require extensive operator experience [5]. Our previous research confirmed that flexible ureteroscopy assisted by a pressure-controlling UAS (PC-UAS) significantly improved safety and efficiency [6].

Therefore, in the present study, we assessed whether rigid ureteroscopic lithotripsy with PC-UAS can achieve favorable results in the treatment of complex steinstrasse syndrome compared with previously published data.

## Materials and methods

### Platform and PC-UAS

**Fig. 1:**
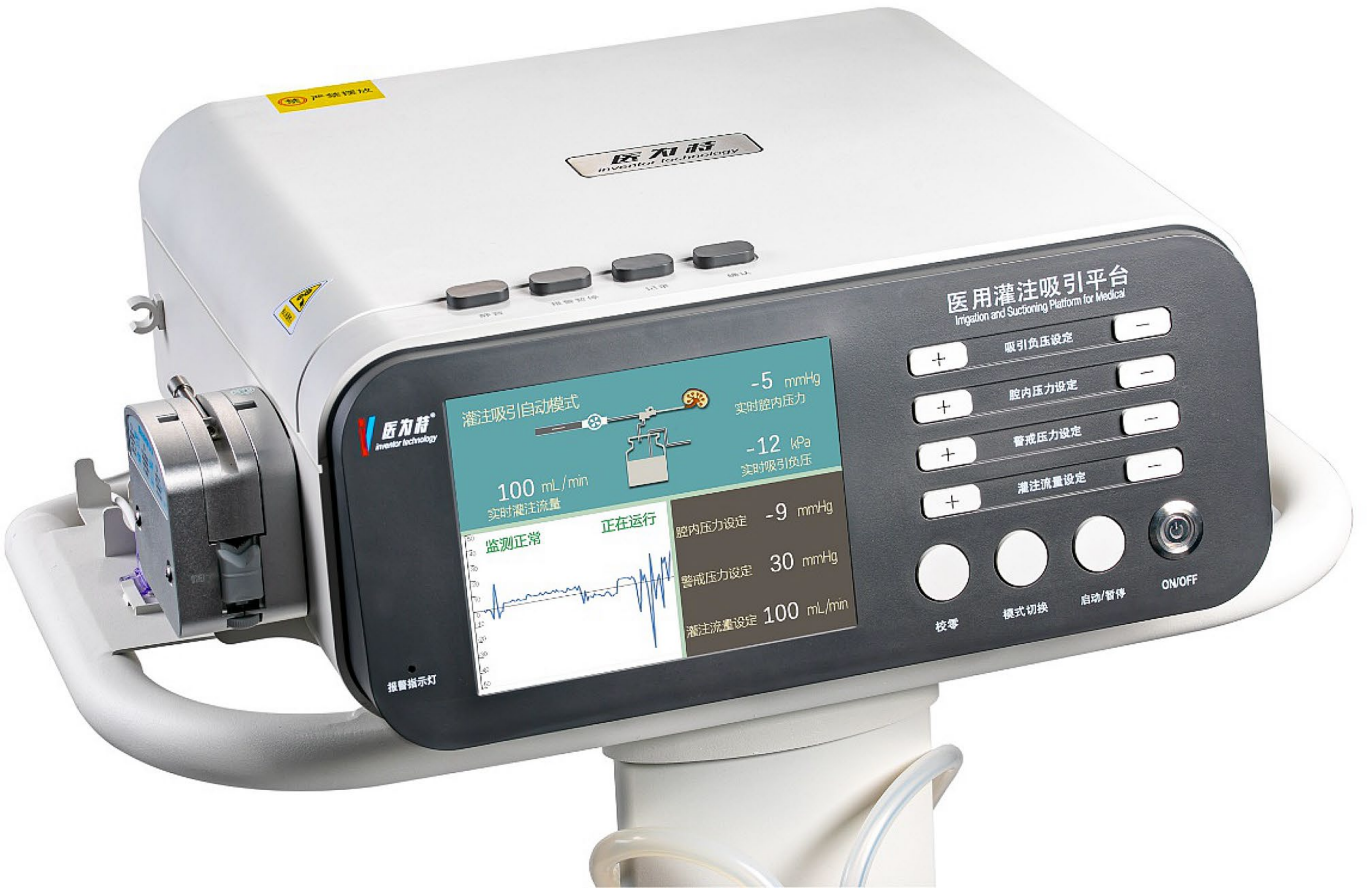
Perfusion and suction platform

**Fig. 2:**
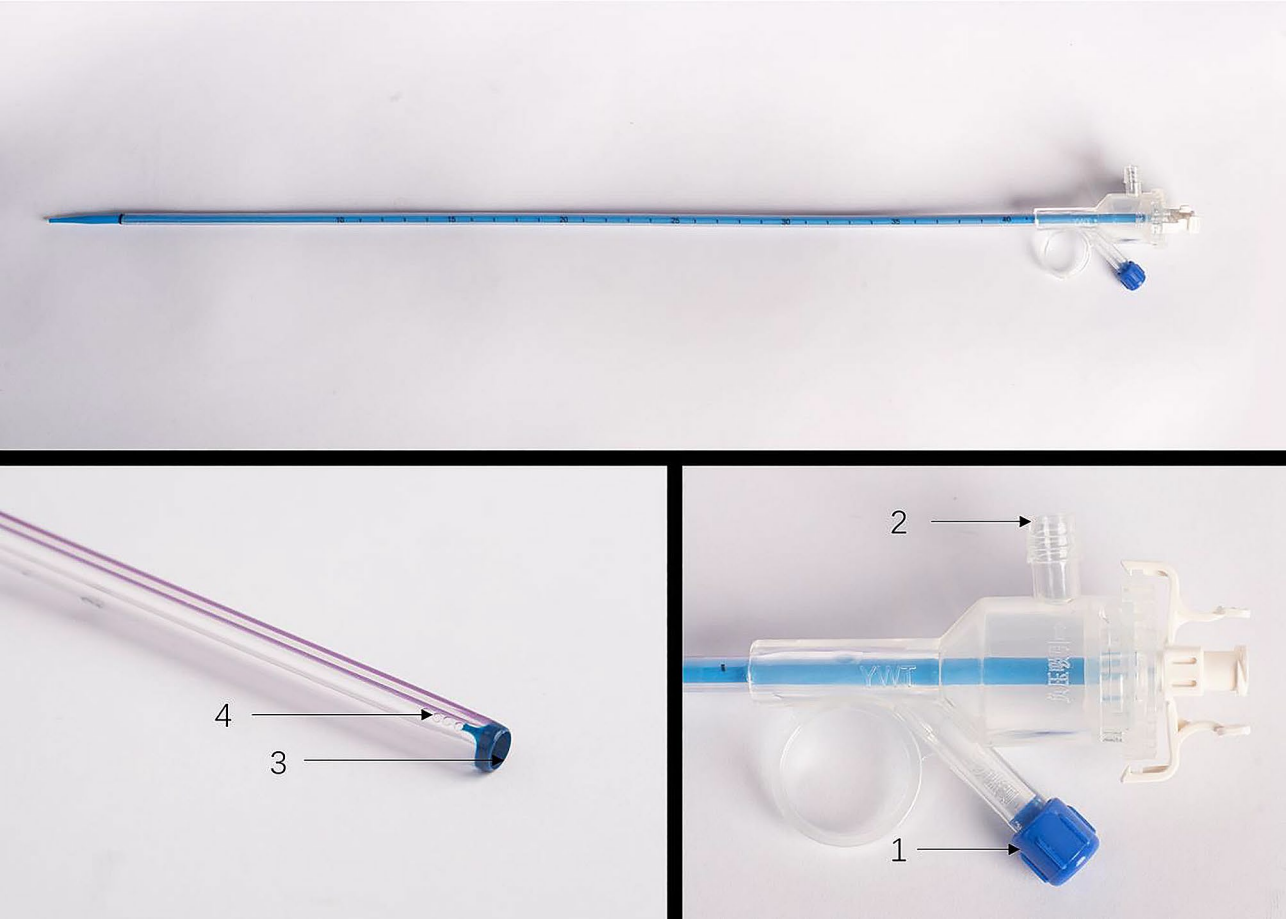
PC-UAS; 1 pressure channel, 2 suctioning channel, 3 working channel, and 4, pressure measuring hole

The system consisted of a perfusion and suction platform (Fig. 1) and a PC-UAS (Fig. 2) (PC-UAS, Inventor Technology, Jiangxi, China). The perfusion and suction platform comprised a main control unit, perfusion device, suction device, and pressure feedback device. Users can set the perfusion flow rate, control pressure value, warning pressure value, and limit value on the platform display. The main control unit of the platform adjusts the suction pressure using pressure feedback. The platform offers two modes: automatic (perfusion, suction, pressure monitoring, and pressure feedback control) and simple. It can display the actual suction and cavity pressures in real-time.

The PC-UAS had an inner diameter of 12 Fr, an outer diameter of 14 Fr, and a length of 35 cm. Its transparent material allows for the direct observation of mucosal conditions through the sheath. The cavity pressure is measured using four pressure holes at the tip. The end of the sheath had two connection channels for suction and pressure monitoring. The suction channel can automatically suck out stones and adjust the pressure, whereas the pressure monitoring channel can monitor the cavity pressure.

### Patients

We retrospectively analyzed data from consecutive patients with steinstrasse between June 2018 and October 2023 at Ganzhou People’s Hospital. The patient inclusion criteria were age 18–80 years and complex steinstrasse containing ≥ 4 stones or with an aggregate length ≥ 1.5 cm [7]. The exclusion criteria were pregnancy, urinary tract abnormalities, kidney malrotation, and positive urine cultures. Steinstrasse was confirmed by imaging, including urinary tract ultrasonography, intravenous urography, and computed tomography (CT). Stone size was calculated using CT. The study was approved by institutional ethics committee of Ganzhou People’s Hospital(TY-HKY2021-012). All patients provided written informed consent prior to surgery.

### Procedural methods

**Fig. 3:**
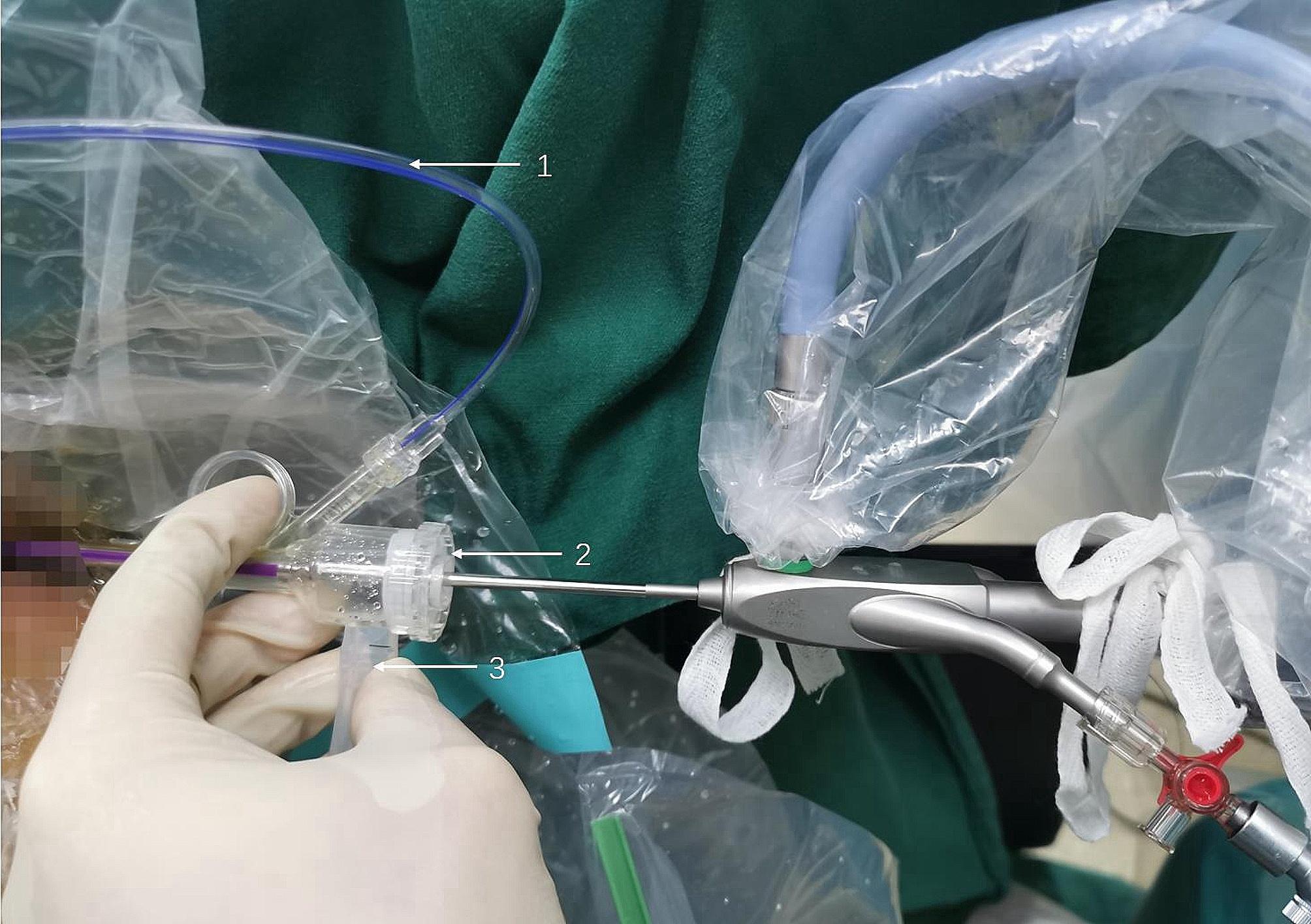
Pipe connection; 1, pressure measuring tube, 2, working channel, and 3, suctioning tube

Rigid ureteroscopic lithotripsy was performed under general anesthesia with the patient in the oblique supine lithotomy position [8]. After the rigid ureteroscope was connected to the irrigation tube, the platform mode was switched to the simple irrigation mode, in which the platform operated similarly to a conventional irrigation pump with an irrigation flow of 50 ml/min. Under the guidance of a guidewire, a rigid 7/8.4 Fr ureteroscope (KARL Storz, Tuttlingen, Germany) was used for ureteroscopy. The rigid ureteroscope had an irrigation channel (2.4 Fr) and a working channel (3.4 Fr). After verifying the absence of ureteral stricture, a guidewire was inserted. The 12/14Fr PC-UAS was placed along the guidewire at the distal end of the stone. The pressure-measuring and suction channels of the sheath were connected to the perfusion suction platform (Fig. 3), and the pressure measuring channel was filled with water and zeroed. The perfusion suction platform was set to fully automatic mode, with a perfusion flow rate of 150 ml/min, control pressure of -15 to -2 mmHg, pressure warning value of 20 mmHg, and limit value of 30 mmHg. During the surgery, a 550 μm diameter fiber was used for rigid lithotripsy with a power of 2.0–2.5 J × 20–30HZ. The broken stone particles were automatically sucked out through the gap between the sheath and the scope, while particles larger than the gap but smaller than the sheath diameter were removed by withdrawing the scope. After surgery, a 4.6 Fr double J was routinely placed for 4 weeks. For patients with ureteral stenosis detected during ureteroscopy, the surgery was changed to ordinary ureteroscopy, and the stones were pushed into the kidneys for PCNL. Laparoscopy was performed for middle and lower ureteral stones that could not be pushed.

After surgery, the patients’ vital signs and blood tests, such as routine blood count, electrolytes, and procalcitonin, were closely monitored. A kidney, ureter, and bladder radiograph (KUB) was performed on postoperative days 1 and 30 to evaluate residual stones. CT scans were performed on patients with X-ray-negative stones. Ultrasonography was performed at 3 months to assess hydronephrosis. Stone-free status was defined as the absence of stone fragments or residual stones < 2mme in CT or < 4 mm on KUB in size. Procedure duration was defined as the time from insertion of the controlled-pressure ureteral suction sheath to the end of the procedure. Surgical complications were graded according to the Clavien-Dindo classification [9].

## Results

**Fig. 4:**
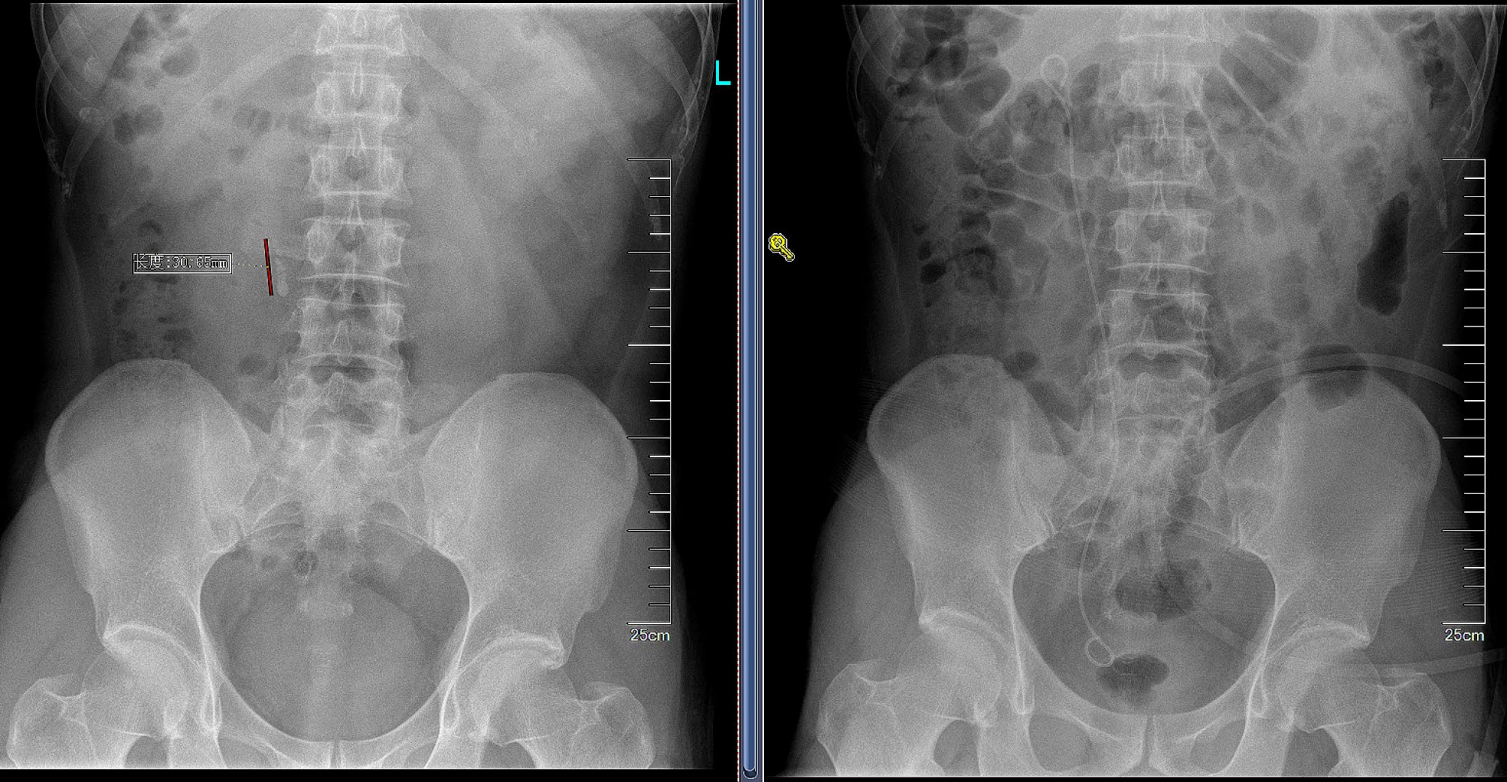
Preoperative and postoperative kidney, ureter, and bladder radiograph (KUB)

A total of 31 consecutive patients were included, six of whom had concurrent kidney stones. Among the included patients, 17 had a history of flexible ureteroscopy, seven had a history of PCNL surgery, five had a history of extracorporeal shockwave, and two had no treatment. In 29 cases, a PC-UAS was successfully inserted, of which nine cases were combined with a flexible ureteroscope, three cases had ureteral stones migrated to the kidneys, and two cases were converted to PCNL due to failure of the sheath insertion. The intraoperative pressure was controlled to within 20 mmHg in all 29 cases, with a clear field of view. There were no cases of ureteral perforation, mucosal avulsion, or hemorrhage during the operation. There were no cases of Clavien-Dindo grades II–V complications. One patient with fever and another with nausea and vomiting recovered after symptomatic treatment. The stone-free rates were 93.1% (27/29) and 96.6% (28/29) on 1 day and 1 month postoperatively, respectively ((Table 1; Fig. 4). The double J was removed successfully 1 month postoperatively, and one patient with residual stones was cleared after an extracorporeal shock wave. At the 3-month follow-up, hydronephrosis was reduced compared to the preoperative period, and there was no perinephric fluid or hematoma formation. Calculus composition analysis revealed 16 calcium oxalate calculi, two calcium phosphate calculi, three uric acid calculi, and eight mixed calculi.

**Table 1:** Basic characteristics and complications of two groups

Variables	PC-UAS group	Control group^[4]^
Age(years)	42.7 ± 14.3	52.2 ± 10.0
Sex		
Male	18	21
Female	13	34
BMI(kg/m^2^)	19.5 ± 1.8	-
Stone size(cm)	2.7 ± 1.3	3.6 ± 2.1
CT value (Hu)	874.6 ± 276.4	-
Position		
Upper	13	15
Middle	9	8
Lower	5	9
Mixed	4	3
UAS placement success rate	93.5% (29/31)	91.4% (32/35)
Operation time(min)	48.2 ± 17.7	33.7 ± 12.2
Cavity pressure (mmHg)	-4.6 ± 2.1	-
Postoperative hospital stay(d)	1.7 ± 0.3	-
Stone-free rates		
1d	93.1% (27/29)	77.1% (27/35)
30d	96.6% (28/29)	82.9% (29/35)
Complications *n* (%)		
Clavien grade I	2(6.9%)	5(14.3%)
Fever	1(3.4%)	2(5.7%)
Nausea/Vomiting/Hematuria	1(3.4%)	3(8.6%)
Clavien grade IV Sepsis	0	1(2.9%)

The data of the control group were obtained from the previously published literature ^[4]^

## Discussion

The surgical management of steinstrasse, especially complex steinstrasse, has always been a difficult problem for urologists. Surgical intervention should be initiated as early as possible in patients who have not been drained within 4 weeks of conservative medication, especially in type II or III steinstrasse [7]. Currently, flexible ureteroscopy is the preferred surgical intervention for steinstrasse stenosis, and high cavity pressure of renal often occurs during surgery [10]. When the cavity pressure exceeds 30–35 mmHg, it leads to reflux of the renal pelvic lymph nodes and veins, which causes the absorption of fluids, bacteria, and endotoxins into the bloodstream, leading to fever, systemic inflammatory response syndrome, sepsis complications, and long-term renal impairment [11, 12]. Impairment of renal function owing to elevated pressure has a cumulative effect [13]. In the case of a complex steinstrasse, the efficiency is low, the operative time tends to be long, and the incidence of infection-related complications is high. Therefore, scholars have reported the use of a suctioning UAS to improve the safety and efficiency of complex steinstrasse [4]. However, this method cannot monitor the cavity pressure of, and there is still a risk of high cavity pressure. To resolve the contradiction between irrigation flow and cavity pressure, in 2015, we developed a flexible ureteroscope with automatic pressure control using a PC-UAS, which achieved better results during clinical use [14, 15]. This system intelligently adjusts the suction pressure based on the pressure value collected by the sheath and feeds it back to the main control unit, thereby maintaining the cavity pressure at a set value. If a blockage caused by stone powder or blood clots causes a rapid pressure increase that exceeds the warning pressure value, the platform sounds an alarm. If the cavity pressure exceeds the limit value, the platform automatically stops perfusion to prevent a high cavity pressure [16].

The combined use of a rigid ureteroscope and PC-UAS for complex steinstrasse has the following advantages. First, it can monitor and automatically control pressure, which reduces fluid absorption and infection complications related to high cavity pressures [17]. Secondly, the rigid ureteroscope is easy to operate and can be used with a 550 μm fiber to significantly improve the efficiency of holmium laser lithotripsy. The 150–200 ml/min perfusion flow carried by the rigid ureteroscope reduces the thermal effect, ensures clear vision, and accelerates stone extraction [18]. The system can rapidly and automatically suction stone fragments without a stone basket, thereby reducing postoperative stone residue and stone migration. In this study, there were two cases of postoperative Clavien-Dindo grade I complications and no cases of Clavien-Dindo grade II–V complications. The complication rate was lower than that of control group using vacuum-assisted ureteral access sheath. No cases of ureteral perforation, mucosal avulsion, or hemorrhage were observed. The operation time of 48.2 ± 17.7 min was significantly shorter than that of traditional flexible ureteroscopy or rigid ureteroscopy [19]. However, the operation time was longer than that of rigid ureteroscopy using vacuum-assisted ureteral access sheath. Stone-free rates of 1 day and 30 day is higher than than that of control group. These are most likely attributable to the effective control of cavity pressure at high perfusion flow. This suggests that this technique can effectively control cavity pressure, shorten the operation time, improve lithotripsy efficiency, and reduce the complication rates.

This study has some limitations. It was a retrospective study and the sample size was small. Due to lack of a concurrent control group and the control group was obtained from the published data, we could not determine the difference in safety and efficacy compared with traditional technique. Also, because of the short follow-up time, we were unable to analyze the incidence of ureteral stricture. Finally, we did not check all CT scans during postoperative follow-up, which may lead to bias in stone free rate. In conclusion, this preliminary study confirms that ureteroscopic lithotripsy with PM-UAS is a safe and effective method for treating complex steinstrasse. To address the study limitations, prospective comparative studies are necessary in the future.

## Data Availability

The data is contained within the manuscript, any missing details will be available from the corresponding author on reasonable request.

## Abbreviations

CT: Computed tomography
KUB: Kidney, ureter, and bladder radiograph
PCNL: Percutaneous nephrolithotomy
PC-UAS: Pressure-controlling ureteral access sheath
UAS: Ureteral access sheath
